# Innate Immunity in the Cottonmouth Watersnake (*Agkistrodon piscivorus*)

**DOI:** 10.3390/ani15213223

**Published:** 2025-11-06

**Authors:** Mark Merchant, Justin Epperson, Sarah Baker

**Affiliations:** 1 Department of Chemistry and Physics, McNeese State University, Lake Charles, LA 70609, USA; 2 Department of Chemistry, McNeese State University, Lake Charles, LA 70609, USA; 3 Department of Biology, McNeese State University, Lake Charles, LA 70605, USA

**Keywords:** serum complement, ectotherm, immune system, pit viper, snakes, squamates

## Abstract

**Simple Summary:**

Reptiles play an important role in nature, but unlike mammals, their immune systems are not well understood. This study examined the cottonmouth snake, a venomous species found in the southeastern United States, to better understand how it defends itself against infection. We found that the snake’s blood has strong natural defenses, able to kill harmful bacteria and break down foreign cells very effectively. In fact, its defensive power was far stronger than that found in other ectothermic animals. This study also identified specific proteins in the blood that may be responsible for these protective effects. These findings are valuable because they help fill a major gap in knowledge about reptile health and survival. Understanding how reptiles fight infection could also inspire new medical research, potentially leading to improved treatments for human and animal diseases.

**Abstract:**

Despite their ecological importance and unique evolutionary history, reptiles remain underrepresented in immunological research. The innate immunity of the cottonmouth (*Agkistrodon piscivorus*), a semi-aquatic pit viper native to the southeastern United States, was characterized to provide insight into the molecular and cellular mechanisms underlying its first line of defense against pathogens. Plasma collected from wild *A. piscivorus* exhibited strong antibacterial activities against both Gram-negative and Gram-positive bacteria. In addition, plasma from *A. piscivorus* showed potent hemolytic activities in unsensitized sheep red blood cell (SRBC) hemolysis assays. This activity was concentration-, time-, and temperature-dependent. In addition, the hemolytic activity was inhibited by mild heat treatment (56 °C, 30 min) of plasma and proteases and also by EDTA, suggesting that the hemolytic activity was due to the presence of serum complement proteins. SDS-PAGE analysis of plasma proteins isolated from a mannan-agarose affinity column revealed the presence of a protein with a mass of 36 kDa, raising the strong possibility that the lectin pathway of complement activation is active. The EC_50_ for hemolysis of SRBCs by plasma from *A. piscivorus* was approximately 10–100× lower than that of any other reptilian species described. This is the first study to characterize innate immunity in *A. piscivorus*.

## 1. Introduction

Eukaryotic organisms must produce a strong immune response to ensure protection from potentially pathological microorganisms. This is achieved through the efforts of the host organism’s immune system. There are two branches of the immune system, namely adaptive and innate immunity. Adaptive immunity provides specific defense against previously exposed microorganisms and allows for an immunological memory, but the responses develop more slowly and can require days to develop and coordinate a defense against a specific microorganism. In contrast, innate immunity provides a non-specific, often rapid response to colonization by microorganisms and does not require previous exposure. Innate immune defenses rely on physical barriers like skin and mucous membranes, as well as immune cells such as phagocytes and natural killer cells, and soluble mediators such as cytokines and enzyme systems such as lysozyme and serum complement proteins [1]. Adaptive immunity involves lymphocytes like B and T cells that recognize unique antigens and produce antibodies that recognize and bind antigens to establish immunological memory [2]. It is thought that adaptive immunity is a more recent immunological development which likely first arose in jawed vertebrates (gnathostomes) [3]. Whereas innate immunity is thought to have originated in more ancient taxa with supporting evidence of innate immune activity found in invertebrates such as sea urchins [4] and even in some plant species [5].

Ectothermic vertebrates rely more on innate immunity than adaptive mechanisms [6,7,8]. A key component of this system is the serum complement pathway, a set of liver-derived plasma proteins that circulate in inactive form and are activated through proteolytic cascades when encountering pathogens [9]. These proteins coordinate to neutralize microbes by promoting inflammation, opsonization, and direct lysis via the membrane attack complex (MAC). Complement activation also generates anaphylatoxins (C3a, C5a), which recruit leukocytes to sites of infection. Complement can be initiated through three pathways—classical, alternative, and lectin—all of which converge on C3 activation and ultimately MAC formation. The classical pathway is triggered by antibody–antigen complexes binding C1. The alternative pathway relies on spontaneous C3 hydrolysis or direct C3b binding to microbial surfaces, producing a distinct C3 convertase that amplifies the response. The lectin pathway is initiated when mannose-binding lectin (MBL) or ficolins bind microbial carbohydrates, activating MASPs that generate the same C3 convertase as the classical route [9]. Bacterial cell wall polysaccharides such as N-acetylglucosamine can facilitate this recognition [10]. Together, these pathways provide a rapid, versatile defense that enhances inflammation, promotes phagocytosis, and lyses target cells.

Interactions between innate and adaptive immunity are crucial for an effective immune response [11]. Innate immune cells, such as dendritic cells, process and present antigens to adaptive immune cells, thereby initiating and shaping the adaptive response. This crosstalk ensures a coordinated defense, where the rapid response of innate immunity informs and enhances the specificity of adaptive immunity, leading to a more robust and efficient protection against pathogens [12]. This interaction between innate and adaptive immunity involves crosstalk between these two arms: for instance, antigen-presenting cells such as dendritic cells bridge the systems by detecting pathogens via innate receptors and activating adaptive responses through antigen presentation and co-stimulatory signaling [13]. This coordinated interaction ensures an efficient and robust defense tailored to a wide range of microbial threats.

While extensively studied in mammals, the complement system of ectothermic vertebrates—including fish, amphibians, and reptiles—has garnered increasing attention due to its evolutionary significance and adaptations to diverse environmental conditions [14]. Ectotherms exhibit complement activity that is temperature-dependent, influencing immune responses in variable climates [15]. Studies with teleost fish have demonstrated that their alternative complement pathway plays a dominant role in pathogen defense, given the absence of immunoglobulin-mediated responses at early developmental stages [16]. Similarly, amphibians like *Xenopus laevis* show a functional complement system that bridges innate and adaptive immunity [17]. Reptiles, though less studied, have been found to express functional complement proteins with potent antimicrobial activities, though their regulation and efficiency remain under investigation [18].

The cottonmouth (*Agkistrodon piscivorus*) is a semi-aquatic pit viper native to the southeastern United States, ranging from southeastern Virginia southward through Florida, and west into central Texas and eastern Oklahoma [19,20,21]. Ecologically, cottonmouths are generalist predators feeding on fish, amphibians, reptiles, birds, and small mammals [20,22,23]. They are strongly associated with aquatic and semi-aquatic habitats such as swamps, marshes, floodplain forests, and slow-moving streams [20,21,22]. Home-range size varies with sex and reproductive status, with males generally ranging farther than females and gravid females showing expanded movements compared to non-gravid females [24]. Movement is typically constrained near water, with the linear structure of riparian habitats shaping spatial ecology [24].

Understanding the complement system in ectotherms is essential for elucidating the evolution of vertebrate immunity and its adaptation to environmental pressures. The goal of this study is to characterize innate immunity and, in particular, the serum complement system, of the cottonmouth watersnake (*Agkistrodon piscivorus*, Lacépède, 1789).

## 2. Materials and Methods

Biochemicals—Nutrient broth and nutrient agar were purchased from ISC Bioexpress (Kaysville, UT, USA). Lyophilized bacterial strains were purchased from ATCC (Manassas, VA, USA). The following-registered strains were used: *Vibrio vulnificus* (27562), *Aeromonas hydrophila* (35654), *Streptococcus pyogenes* (19615), and *Staphylococcus aureus* (6538). Protease derived from *Streptomyces griseus*, EDTA, methylamine, and salicylaldoxime were purchased from Sigma Chemical Company (St. Louis, MO, USA).

Treatment of Animals–Adult snakes (≥60 cm total length, sex and reproductive status not determined) were captured at McFaddin National Wildlife Refuge (near Sabine Pass, Texas) during the months of May-August and secured in transparent polypropylene cylindrical tubes for safe handling. Whole blood (0.5–2.0 mL) was collected via the caudal vein from eight snakes using 1.77 cm, 26-gauge needles [25]. The blood was transferred to heparinized blood collection tubes and pooled. Plasma was isolated by centrifugation at 2500× *g* for 5 min and then stored at −20 °C until used for assays.

Antibacterial Assays—Two-mL samples of sterile nutrient broth containing various dilutions of plasma derived from *A. piscivorus* were inoculated with approximately 1 × 10^4^ of each bacterial species (20 μL) from a log phase culture. Aliquots (150 μL) of each culture were transferred to wells, in quadruplicate, in 96-well microtiter plates and incubated for 12 h at 37 °C. The optical density of each sample was measured at 0, 3, 6, 12, and 24 h using a BioRad (Hercules, CA, USA) Benchmark Plus™ microtiter plate reader spectrophotometer at 430 nm. Baseline absorbance values were recorded for each well, and subsequent readings at each time point were compared to their respective baselines.

Serum Complement Activity—Serum complement activity in *A. piscivorus* plasma was assessed using a SRBC hemolysis assay modified from the method of Mayer (1967). Plasma collected from wild *A. piscivorus* (200 μL) was incubated with an equal volume of 2% SRBCs (*v*/*v*) for 30 min at ambient temperature and then centrifuged at 2500× *g* for 3 min. The supernatant (150 μL) was removed to a 96-well plate, and the optical density was measured at 540 nm in a Benchmark Plus microtiter plate reader (BioRad, Hercules, CA, USA). To determine the titer-dependent effects of snake plasma on hemolysis, 200 μL of different volumes of plasma (0, 200, 400, 600, 800, and 1000 μL) diluted in saline was incubated with 200 μL of 2% SRBCs for 30 min at ambient temperature. The SRBCs were centrifuged, and the optical density of the supernatant was measured as described above. The CH_50_ for hemolysis was calculated by the method of Fike [26]. For the kinetic hemolysis analysis, 7.5 mL of either 1% or 2% snake plasma, diluted in normal saline, was mixed with 7.5 mL of 2% SRBCs. Aliquots were removed at various time points (0, 2, 5, 10, 15, 20, 30, and 60 min) and immediately centrifuged at 2500× *g* for 3 min. The supernatants (150 μL) were removed to a 96-well plate, and the optical density was measured at 540 nm as described above.

Undiluted plasma from *A. piscivorus* incubated with or without saline and 20 mM ammonium hydroxide, 20 mM methylamine, 20 mM salicylaldoxime, or 5 mM EDTA for 30 min at room temperature. The serum samples were then incubated with 1% SRBCs for 30 min. Samples were centrifuged at 1500× *g* and the optical densities of the supernatants were determined at 540 nm. The results are expressed as the maximum of the positive sample control sample lysed with a TB syringe in the presence of 1% Triton X-100 detergent. In addition, plasma samples were also treated with 5 mM EDTA with the addition of either 20 mM CaCl_2_ or 20 mM MgCl_2_.

Lectin Isolation—Proteins were isolated from *A. piscivorus* plasma with affinity chromatography. Mannan agarose (3 mL, Sigma-Aldrich, St. Louis, MO, USA) was equilibrated with 3 mL of mixing buffer (10 mM Tris-HCl, pH 7.8, 1.25 M NaCl) and allowed to settle into a column prepared from a Pasteur pipette plugged with cotton. Fresh *A. piscivorus* plasma (5 mL) was mixed with 5 mL of loading buffer (20 mM Tris-HCl, pH 7.8, 2.5 M NaCl). The diluted serum was allowed to filter through the column and the column was washed with 10 volumes of loading buffer. The proteins were eluted with 5 mL of elution buffer (10 mM Tris-HCl, pH 7.8, 1.25 M NaCl, 2 mM EDTA). The isolated lectin protein was transferred to 10 kDa microcentrifugal concentrator tubes (Centriprep, Millipore Corp., Billerica, MA, USA) and centrifuged at 4500× *g* to concentrate and desalt the sample, and 35 mL of the concentrated sample was used for SDS-PAGE mass analysis.

SDS-PAGE—One-dimensional SDS-PAGE was performed using pre-cast 4–20% polyacrylamide gradient tris-HCl gels on a small format gel electrophoresis system (Criterion, Bio-Rad). The samples were mixed 1:1 *v*/*v* with sample buffer containing 62.5 mM tris-HCl at pH 6.8, 2% SDS, 25% glycerol (*w*/*v*), 0.01% *w*/*v* bromophenol blue and in both the presence and absence of 10% 2-mercaptoethanol (*v*/*v*) and heated at 95 °C in a water bath for 5 min before loading on the gel. Samples were electrophoresed at 40 V for 3 h using tris-glycine (pH 8.3) as the gel running buffer to acquire high resolution separation of proteins. Gels were stained with Coomassie blue for 1 h and rinsed twice with distilled water for 10 min each time to remove stain. The gel images were captured using the *Gel* Doc XR System and with Quantity One 1-D analysis software (BioRad, Hercules, CA, USA).

Statistics and controls—All experiments were performed in quadruplicate to obtain valid statistical evaluation of the results, and all results represent the means ± S.D. CFUs/mL for each sample were calculated by multiplying the number of colonies counted by the dilution factor and then by 50 (due to the fact that only 20 µL were plated on each dish). For each SRBC hemolysis assay, a complete lysis positive control was obtained by rapidly syringing a suspension of 1% (*v*/*v*) SRBCs in water through a 31 ga needle. This action resulted in 100% hemolysis, as confirmed by inspection under 400× magnification under a phase contrast microscope. The spectrophotometric results of incubation of SRBCs with isotonic saline were subtracted from the results obtained from each sample, thereby removing the small effects of autohemolysis. Statistical analysis was conducted in R software, v 3.5.1 [27] at an alpha level of 0.05.

## 3. Results

**Figure 1:**
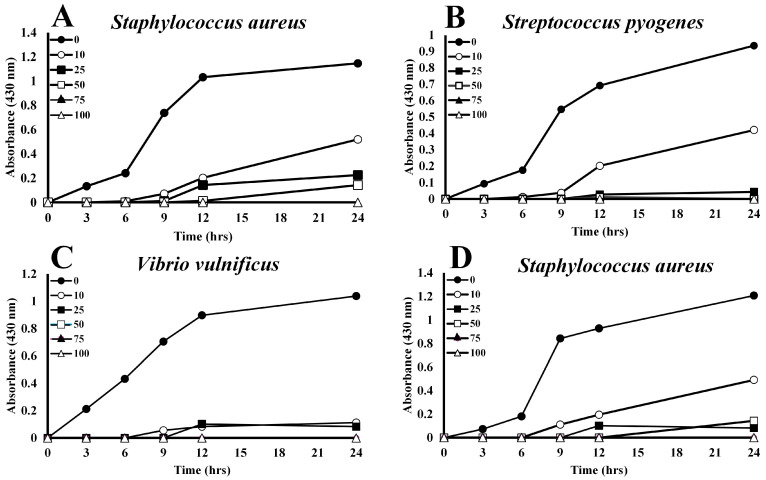
Concentration-dependent inhibition of bacterial growth by *A. piscivorus* plasma. Culture tubes containing 2 mL of 0, 10, 25, 50, 75 or 100% plasma from *A. piscivorus* were inoculated with 10^6^ CFU different bacterial species and 150 mL were transferred to microtiter plates. Plates were incubated at 37 °C, and their optical densities were monitored (430 nm) at 0, 3, 6, 12, and 24 h post-inoculation. The results are expressed as the means ± standard deviations. for four independent determinations.

The data displayed in Figure 1 shows the volume-dependent growth reduction in Gram-positive bacteria by plasma derived from *A. piscivorus*. Inoculation of nutrient broth with a final titer of approximately 1.7 × 10^6^ bacteria produced robust growth.; however, inclusion of only 10% plasma (*v*/*v*) inhibited the growth of *Staphylococcus aureus* by 54.7% at 24 h (Figure 1A) and increases to 25 and 50% plasma resulted in 80.4 and 87.5% reductions in growth, respectively. Higher concentrations (75 and 100%) of plasma completely inhibited the growth of *S. aureus* (Figure 1A). Incubation of *S. pyogenes* with 10% *A. piscivorus* plasma produced a 59.3% decrease in growth at 24 h (Figure 1B), while 25 and 75% plasma inhibited growth by 93.1 and 88.1%, respectively. Furthermore, the inclusion of 75 and 100% plasma reduced bacterial growth to undetectable levels.

Low levels of plasma from *A. piscivorus* also inhibited the growth of Gram-negative bacterial species. Growth of *V. vulnificus* was inhibited by 89.2% by the addition of only 10% plasma (Figure 1C). Growth was reduced by 92.0% with 25% plasma, and 50–100% plasma entirely prevented bacterial growth. Furthermore, 10 and 25% plasma from *A. piscivorus* impeded growth of *A. hydrophila* by 55.0% and 95.7% at 24 h (Figure 1D), respectively, while 50, 75, and 100% plasma completely inhibited bacterial growth.

**Figure 2:**
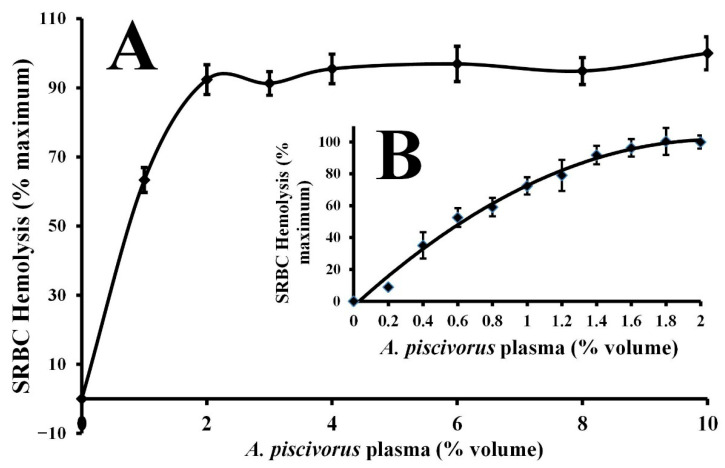
Volume-dependent hemolysis of SRBCs by plasma derived from *A. piscivorus*. (**A**) Different volumes (0–10% volume, in 1–2% increments) of pooled plasma samples were diluted in sterile saline and incubated with 1% SRBCs for 30 min at ambient temperature. (**B**) Different volumes (0–2% volume, in 0.2% increments) of pooled plasma samples were diluted in sterile saline and incubated with 1% SRBCs for 30 min at ambient temperature Hemolysis activity was determined by spectrophotometry at 540 nm and expressed as % maximum as compared to a complete hemolysis positive control. The data represent means ± standard deviations for four independent determinations.

Incubation of plasma from *A. piscivorus* with 1% (*v*/*v*) SRBCs resulted in hemolysis as determined by increases in absorbance at 540 nm (Figure 2). The hemolytic activity for a broad range of dilutions of *A. piscivorus* plasma is displayed in Figure 2A. Inclusion of only 1% (*v*/*v*) of plasma resulted in 63.3 ± 3.6% hemolysis, and all other concentrations of plasma resulted in hemolysis not statistically different than 100% (*p* > 0.05). A closer examination of the hemolytic activity of plasma between 0 and 2% (*v*/*v*) is shown in Figure 2B. The hemolysis activity increased progressively with increased plasma volume. The EC_50_ for the hemolysis of SRBCs was calculated to be 0.62% plasma (*v*/*v*).

**Figure 3:**
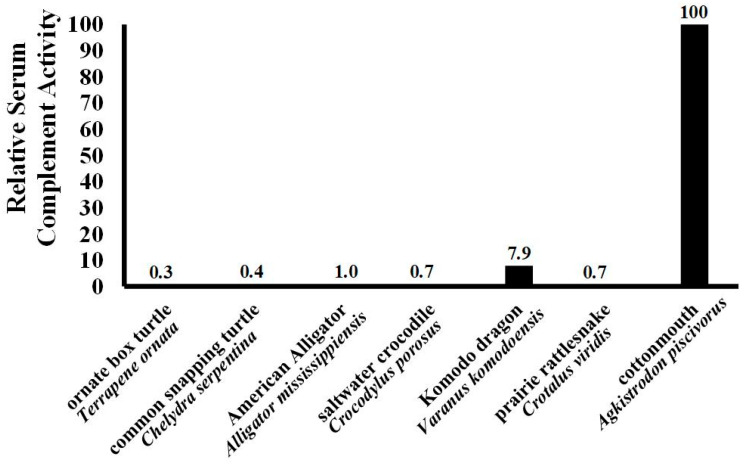
Relative hemolysis of SRBCs among different reptilians. The EC_50_ values of other ectotherms determined during other studies in our laboratory were compared to highlight the strength of the robust complement response observed for *A. piscivorus* in the present study.

**Figure 4:**
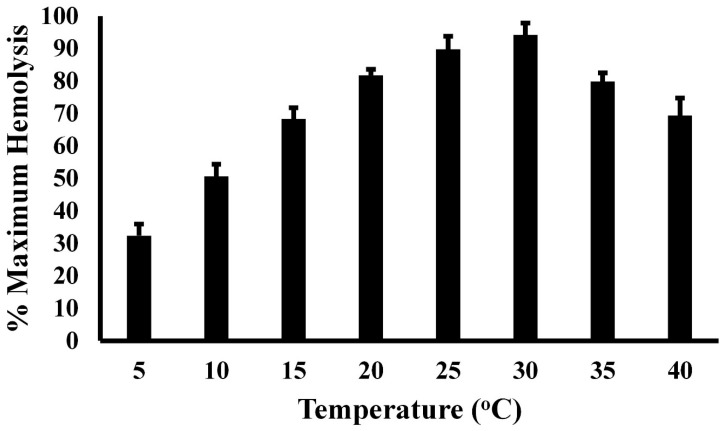
Temperature-dependent hemolysis of SRBCs by plasma from *A. piscivorus*. Pooled plasma samples were diluted in unbuffered saline and incubated with 1% SRBCs at different temperatures. Hemolysis activity was determined by spectrophotometry at 540 nm and expressed as % maximum as compared to a complete hemolysis positive control. The data represent the means ± standard deviations for four independent determinations.

The strong hemolytic response of plasma derived from *A. piscivorus* (Figure 2) exhibited an EC_50_ for the hemolysis of SRBCs that was less than 0.62% plasma (Figure 3). This value is extremely low when compared to other values of other diverse reptiles (Figure 4) such as the American alligator (*Alligator mississippiensis*, EC_50_ = 6.0%) [18], Saltwater crocodile (*Crocodylus porosus*, EC_50_ = 8.67%) [28], prairie rattlesnake (*Crotalus virdis*, EC_50_ = 8.87%) [29], common snapping turtle (*Chelydra serpentina*, EC_50_ = 16.2%) [30], ornate box turtle (*Terrapene ornata*, EC_50_ = 24.4%) [31], and Komodo dragon (*Varanid komodoensis*, EC_50_ = 4.9%) [32].

Incubation of 1% SRBCs (*v*/*v*) with 5% plasma from *A. piscivorus*, diluted in sterile saline, for 30 min. resulted in 98.8 ± 1.5% of maximal hemolysis (Table 1). However, incubation with 5% plasma that had been incubated at 56 °C for 30 min. exhibited low hemolytic activity (2.3 ± 0.4%, *p* < 0.0001). Likewise, incubation of plasma with 20 U of protease derived from *S. griseus* resulted in a 98.5% reduction in activity (1.8 0.8%, *p* > 0.0001). Furthermore, the addition of 5 mM EDTA to an incubation of plasma and 1% SRBCs almost completely inhibited hemolytic activity, as hemolysis was reduced to 2.3 ± 0.4% of maximum. However, the inclusion of molar excess of divalent metal ions restored the EDTA-mediated decline in hemolysis, as hemolytic activity was increased to 98.6 ± 1.1% and 97.1 ± 0.9% by the addition of Ca^2+^ or Mg^2+^, respectively. The addition of 20 mM methylamine to plasma prior to incubation with SRBCs did not affect the hemolytic effects of the plasma (98.8 ± 1.2%, *p* > 0.05), while the incubation of 20 mM salicylaldoxime reduced hemolysis by 97.8% (2.2 ± 0.5%, *p* > 0.0001). The presence of EDTA, methylamine, or salicylaldoxime in the absence of plasma did not affect the integrity of the SRBCs and thus no hemolysis was observed.

**Figure 5:**
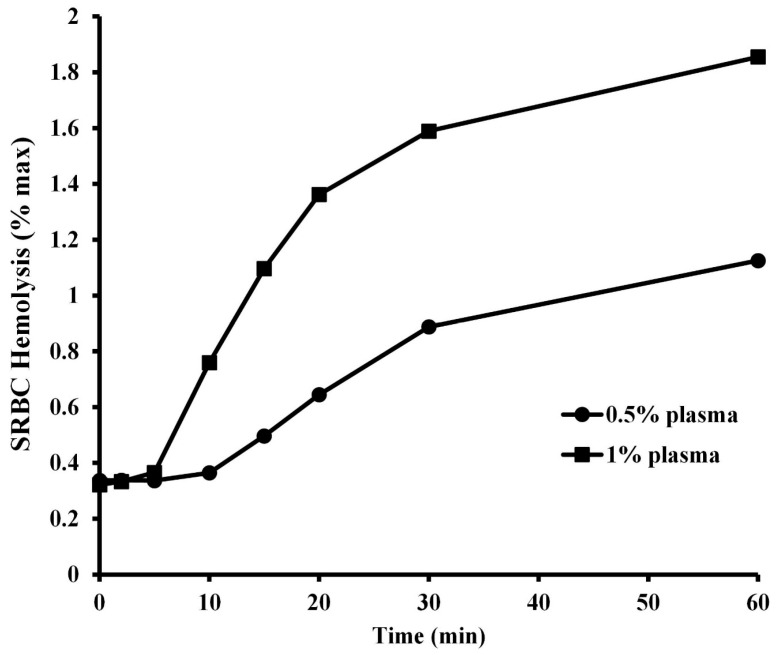
Kinetic analysis of SRBC hemolysis by plasma from *A piscivorus*. Pooled plasma samples were diluted in unbuffered saline were incubated with 1% SRBCs for different amounts of time. Hemolysis activity was determined by spectrophotometry at 540 nm and expressed as % maximum as compared to a complete hemolysis positive control. The data represent the means ± standard deviations for four independent determinations.

The kinetic character of the hemolysis of SRBCs by two different concentrations of *A. piscivorus* plasma is illustrated in Figure 5. Incubation of 1% (*v*/*v*) SRBCs with 1% (*v*/*v*) plasma resulted in a lag period of 10 min. during which no activity was observed (*p* > 0.5) followed by a period of rapid hemolysis from 10 to 20 min. and a slower rate of increase in hemolysis at 60 min. (95.6 ± 1.8%). Likewise, incubation of SRBCs with 0.5% plasma resulted in a lag period of no hemolysis (*p* > 0.5) for 15 min., followed by a slow increase to 30 min and a slight increase in activity at 60 min. (66.0 ± 1.3%).

The thermal profile of serum complement activity is illustrated in Figure 4. Plasma derived from *A. piscivorus* showed low serum complement activity at 5 °C (32.3 ± 3.6% of maximal activity), followed by a steady increase from 5 to 25 °C and peak activity at 30 °C (94.2 ± 3.7% of maximal activity). The near-linear relation (R^2^ = 0.989) from 5 to 25 °C resulted in an increase of 2.9% for every 1 °C increase in temperature. The activity at 25 °C (89.7 ± 4.0) and 30 °C (94.2 ± 3.7) were not statistically different (*p* = 0.23). The hemolytic activity then decreased stepwise to 79.8 ± 2.7% (*p* = 0.014) and 69.4 ± 5.4% (*p* = 0.002) at 35 and 40 °C, respectively.

**Figure 6:**
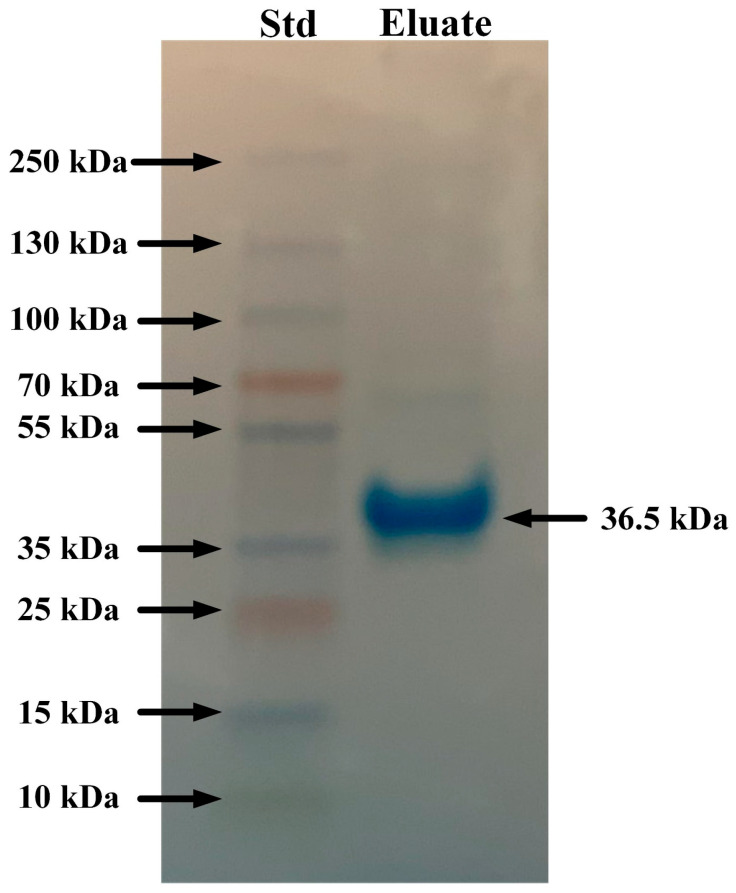
SDS-PAGE analysis of eluates from mannan-agarose affinity column. Plasma samples (5 mL) from *A. piscivorus* were filtered through a mannan-agarose affinity column in the presence of 20 mM Ca^2+^. Samples were eluted using 5 mM EDTA buffer, concentrated and desalted using microcentrifugal concentrators, resolved by SDS PAGE, and stained using Coomassie blue.

Mannan-agarose affinity chromatography coupled with SDS-PAGE analysis showed that *A. piscivorus* expressed high quantities of a protein of approximately 36 kDa (Figure 6). The results from the protein gel also show a very light band at ~70 kDa that near the lower limit of detection, which is most likely a dimer of 36 kDa. The C-type lectin that binds mannan on the surface membranes of microbes is known to dimerize and then form a tetramer in its functional form [33]; this band on the gel is likely an artifact as the SDS and b-mercaptoethanol failed to completely separate the lectin subunits, and this band had been observed in greater quantities during other studies conducted in our laboratory [30,31,33].

## 4. Discussion

Plasma from *A. piscivorus* exhibits strong antibacterial activities at low concentrations against a broad spectrum of diverse bacteria. The inclusion of only 0.5–1% of *A. piscivorus* plasma with 1% SRBCs (*v*/*v*) resulted in rapid hemolysis as determined by increases in A_540_ (Figure 1). *A. hydrophyla* and *V. vulnificus* were chosen because these are bacterial species that are environmentally and ecologically relevant. Watersnakes that inhabit the marshes in the region of this study are constantly exposed to these aquatic bacterial species that can be virulent to snakes [34]. Thus, it is reasonable to expect that wild *A. piscovorus* used in this study would have been exposed to these bacterial species and thus would have antibodies directed towards protein antigens expressed on the surface of these bacteria and thus would have immunity toward these species. However, *S.s pyogenes* and *Staphylococcus aureus* are typically human pathogens, and thus wild *A. piscovorus* would most likely have limited exposure to these bacterial species. However, the potent antibacterial activity of plasma derived from *A. piscovorus* exhibited toward these species showed that the antimicrobial activities are innate in nature.

Based on the EC_50_ data collected from other reptiles (Figure 3), plasma from *A. piscovorus* exhibits comparatively more potent complement activity among other species tested in our laboratory. This indicates it requires the lowest concentration of plasma to achieve 50% of maximum lytic activity, demonstrating significantly higher efficacy compared to the other reptiles. The potency of SRBC hemolysis was approximately 330× stronger than the ornate box turtles (*T. ornata*) [31], 250× stronger than common snapping turtles (*Chelydra serpentina*) [30], 140× stronger than prairie rattlesnakes (*C. viridis*) [29], 100× stronger than American alligators (*A. mississippiensis*) and 12× more potent than the Komodo dragon (*Varanus komodoensis*) [32]. It is not known at this time why plasma derived from *A. piscivorus* exhibits such high hemolytic activity; however, this heightened potency suggests an especially robust innate immune response in *A. piscovorus* relative to both other snakes and reptiles in general. Because these snakes are semi-aquatic and inhabit environments rich in microorganisms, including bacteria, fungi, and parasites found in water and soil, this could exert constant pressure on the immune system to rapidly and effectively recognize and eliminate pathogens. The common snapping turtle and American alligator live in similar habitats, however both have enhanced protection from injury—shell for the snapping turtle and thick skin/osteoderms for the alligator—that limit their risk of a skin injury that could provide an entry point for pathogens. Snakes, lacking the dermal protection of other reptiles, may need to exhibit a more robust immune response to compensate. Previous research has found Lake Erie watersnakes (*Nerodia sipedon insularum*), which are also semi-aquatic, have a relatively robust immune response compared to other reptiles [35], lending support to the suggestion that pathogen presence in aquatic habitats necessitates a stronger innate immune system.

Most immunological proteins are stable at 56 °C, including antimicrobial peptides that interact with, and disrupt, microbial membranes [36]. However, complement proteins are highly thermally labile and easily inactivated at this temperature [37,38,39]. The incubation of plasma from *A. piscivorus* at 56 °C for 30 min reduced the hemolysis of SRBCs dramatically when compared to the activity of untreated plasma. In addition, complement proteins require divalent metal ions (Mg^2+^ and/or Ca^2+^) for activity [40]. For instance, Ca^2+^ is critical for stable interactions between C1q, C1r, and C1 to form the C1 complex [41]. In addition, the formation of the C3 convertase C4b2a in classical/lectin; C3bBb in alternative) requires Mg^2+^ for mammalian complement activity [42], especially for binding of factor B to C3b and also for cleavage of factor B by factor D (in the alternative pathway). These divalent metal ions are also required for complement activity in other reptiles, including *Alligator mississippiensis* [40], *C. viridis* [29], *V. komodoensis* [32], and *Macrochelys temminckii* [30], and *T. carolina carolina* [31]. The hemolysis of SRBCS by plasma from *A. piscivorus* was strongly inhibited by treatment with protease (Table 1), indicating the molecules responsible for the activity were proteinaceous in nature. When EDTA is included in the incubation with SRBCs, the hemolytic activity is reduced drastically, presumably by the sequestration of divalent metal ions away from the complement proteins (Table 1). However, when a molar excess of Mg^2+^ or Ca^2+^ ions are added to the incubation, the divalent metal ions overcome the negative effects of the EDTA and plasma-mediated hemolysis is restored. In addition, the addition of 20 mM salicylaldoxime drastically reduced hemolysis. Salicylaldoxime has been shown to inhibit the alternate complement pathway [43]. The inclusion of 20 mM methylamine did not affect the plasma-mediated hemolysis of SRBCs (Table 1), and this compound is known to interfere with the activity of the C4 protein and inhibit classical complement activity [44,45]. Taken together, the heat inactivation and requirement for divalent metal ions provide overwhelming evidence that the hemolysis of SRBCs is mediated by the serum complement system of proteins. The potent complement activity observed in this study (Figure 2) is probably responsible for the majority of the strong antibacterial activities exhibited by the plasma (Figure 2), particularly since the mechanistic studies suggest that the activities are susceptible to proteases, heat labile, require Mg^2+^ or Ca^2+^ (Table 1), and are very active against bacterial species that are probably not encountered often in the field. This is probably a result of alternative activity that does not require previous exposure antibody–antigen interactions, or a result of mannan-binding lectins that activate the lectin pathway (Figure 6).

The kinetic curves of SRBC hemolysis with two different dilutions of plasma both show an initial lag period followed by a rapid increase in hemolysis, and then a plateau phase as the reaction reaches saturation. The sigmoidal shapes of the curves are indicative of non-Michaelis-Menten cooperative interactions between the complement proteins. This cooperativity of serum complement components has also been observed in a broad spectrum of amphibians and reptiles, including the three-toed amphiuma (*Amphiuma tridactylum*) [46] common snapping turtle (*C. serpetina*) [30], ornate box turtle (*T. ornata*) [31], Komodo dragon (*V. komodoensis*) [32], and prairie rattlesnakes (*C. viridis*) [29].

The evolution of innate and adaptive immunity in endotherms and ectotherms, respectively, has been shaped by distinct energetic constraints. Endotherms, which maintain a stable internal temperature through metabolic heat production, invest heavily in adaptive immunity, which includes memory-based responses that require substantial energy for lymphocyte proliferation, antibody production, and long-term maintenance of immunological memory [47]. In contrast, ectotherms, which rely on external temperatures for thermoregulation, predominantly utilize innate immunity due to its lower metabolic cost and immediate responsiveness [48]. The energetic demands of adaptive immunity may be limiting for ectotherms, especially in fluctuating environments where metabolic rates vary with environmental temperature [49]. Conversely, the high metabolic rates of endotherms enable them to sustain adaptive immune responses, which confer long-term protection and enhanced survival against recurrent infections, justifying the higher energetic investment. Thus, immune system evolution is closely tied to thermoregulatory strategy and energy availability. The thermal profile of complement activity is illustrated in Figure 4. The fact that serum complement activity is compromised at elevated temperatures could mean that these ectotherms may face challenges in the face of rising global temperatures.

The interaction of a mannan-agarose column with plasma from *A. piscivorus* resulted in the elution of a protein of approximately 36 kDa (Figure 6). The fact that the protein was eluted by the addition of EDTA implies that this protein is a C-type lectin that is involved in innate immunity [50]. Mannan-binding lectin (MBL), also known as mannose-binding lectin, is a crucial pattern recognition molecule in the innate immune system of vertebrates [51,52]. It recognizes and binds to carbohydrate motifs such as mannose, fucose, and N-acetylglucosamine that are commonly found on the surfaces of a wide range of pathogens, including bacteria, viruses, fungi, and parasites. Once bound, MBL initiates the lectin pathway of complement activation by associating with MBL-associated serine proteases (MASPs), particularly MASP-2, leading to the cleavage of complement components C4 and C2 and formation of the C3 convertase [53]. This activation promotes opsonization, enhances phagocytosis, and triggers the membrane attack complex that lyses target cells. In addition to its role in complement activation, MBL modulates immune responses by facilitating the clearance of apoptotic cells and influencing cytokine production [54]. Overall, MBL is a vital component of first-line defense in vertebrate immunity, linking pathogen recognition to complement-mediated clearance. The presence of this protein in plasma from *A. piscivorus* is most likely responsible for the antibacterial activity (Figure 1) that is probably mediated by complement activation which leads to lysis of bacterial cells.

## 5. Conclusions

The plasma of cottonmouths (*A. piscivorus*) exhibits potent antibacterial properties that are likely mediated by serum complement-mediated lysis of microbes. The serum complement activity is concentration-, time-, and temperature-dependent and is inactivated by mild thermal treatment and chelators of divalent metal ions. In addition, the plasma contains high concentrations of a C-type lectin that binds mannan, and thus, these findings suggest that all three mechanisms of complement activation (classical, alternative, and lectin-mediated) may be active in *A. piscivorus*. Because only eight snakes were sampled from one season, more work should be conducted to determine if sex- or maturity-dependent or seasonal variations in the strong complement activity exist.

## Figures and Tables

**Table 1:** Effects of Serum Complement Modulators on Hemolysis of SRBCs by Plasma from *A. piscivorus*.

Plasma Treatment	SRBC Hemolysis (% Maximum)
None	98.8 ± 1.5
20 U protease	1.5 ± 0.8 **
5 mM EDTA	2.3 ± 0.4 **
5 mM EDTA + 20 mM Ca^2+^	98.6 ± 1.1 *
5 mM EDTA + 20 mM Mg^2+^	97.1 ± 0.9 *
20 mM methylamine	98.8 ± 1.2 *
20 mM salicylaldoxime	2.2 ± 0.5 **

Plasma was incubated with undiluted plasma from *A. piscivorus* incubated with or without saline and ammonium hydroxide, methylamine, salicylaldimine, or EDTA for 30 min at room temperature. The results represent means ± standard deviations for four determinations. * = not statistically different from treatment with *A. piscivorus* serum alone (*p* > 0.05); ** = statistically lower than treatment with *A. piscivorus* serum alone (*p* < 0.0001).

## Data Availability

Data is available from the corresponding author upon request.
